# Sustaining clinician penetration, attitudes and knowledge in cognitive-behavioral therapy for youth anxiety

**DOI:** 10.1186/s13012-014-0089-9

**Published:** 2014-07-17

**Authors:** Julie M Edmunds, Kendra L Read, Vanesa A Ringle, Douglas M Brodman, Philip C Kendall, Rinad S Beidas

**Affiliations:** 1 Center for Effective Child Therapy, Judge Baker Children’s Center, 53 Parker Hill Avenue, Boston 02120, MA, USA; 2 Department of Psychology, Temple University, 1701 North 13th Street, Philadelphia 19122, PA, USA; 3 New York University Child Study Center, 1 Park Avenue, 7th floor, New York 10016, NY, USA; 4 Department of Psychiatry, University of Pennsylvania, 3535 Market Street, 3rd Floor, Philadelphia 19104, PA, USA

**Keywords:** Sustainment, Evidence-based practice, Training, Consultation, Knowledge, Attitudes, Implementation, Penetration

## Abstract

**Background:**

Questions remain regarding the sustainment of evidence-based practices following implementation. The present study examined the sustainment of community clinicians’ implementation (*i.e*., penetration) of cognitive-behavioral therapy, attitudes toward evidence-based practices, and knowledge of cognitive-behavioral therapy for youth anxiety two years following training and consultation in cognitive-behavioral therapy for youth anxiety.

**Methods:**

Of the original 115 participants, 50 individuals (43%) participated in the two-year follow-up. A t- test examined sustainment in penetration over time. Hierarchical linear modeling examined sustainment in knowledge and attitudes over time. Time spent in consultation sessions was examined as a potential moderator of the change in knowledge and attitudes.

**Results:**

Findings indicated sustained self-reported penetration of cognitive-behavioral therapy for anxious youth, with low fidelity to some key CBT components (*i.e*., exposure tasks). Follow-up knowledge was higher than at baseline but lower than it had been immediately following the consultation phase of the study. Belief in the utility of evidence-based practices was sustained. Willingness to implement an evidence-based practice if required to do so, appeal of evidence-based practices, and openness toward evidence-based practices were not sustained. Participation in consultation positively moderated changes in knowledge and some attitudes.

**Conclusions:**

Sustainment varied depending on the outcome examined. Generally, greater participation in consultation predicted greater sustainment. Implications for future training include higher dosages of consultation.

## Background

Sustainment, ‘the continued use of program components and activities for the continued achievement of desirable program and population outcomes’ [1], is an understudied area within implementation science [2],[3]. Fortunately, the burgeoning of prospective implementation trials has poised the field to explore sustainment following implementation [2]. An understanding of sustainment and associated factors can be informed by an ecological perspective, such as the Exploration, Preparation, Implementation, and Sustainment (EPIS) framework [4]. The EPIS is one of the first frameworks to delineate a separate stage for sustainment, as well as offer guidance regarding contextual factors likely to impact sustainment [4].

A seminal review of sustainment found that many naturalistic observational studies (N = 125) have been conducted following implementation of an innovation. However, the authors caution that these studies are hampered by their insufficient methodological rigor, making it difficult to draw conclusions and necessitating more empirical study [2]. Importantly, there are several areas of weaknesses in the studies conducted to date. First, and most strikingly, only 6% of the studies investigated sustainment following experimental manipulation of implementation strategies. Second, less than half of the studies reported quantitative outcomes, such as the proportion of sites or providers sustaining an EBP. Third, of those studies that did report quantitative outcomes, the majority of investigations only examined penetration [2], which refers to ‘the integration of a practice within a service setting and its subsystems’ [5]. Other important sustainment outcomes were not examined. Fourth, when examining predictors of sustainment, only 30 studies used quantitative methods, and of those studies, only 20 were guided by a conceptual framework [2]. Given these limitations, the current study contributes to the empirical literature on sustainment by: investigating sustainment following an experimental manipulation of an implementation strategy (*i.e*., training); examining a number of sustainment outcomes quantitatively (*i.e*., penetration, components of treatment utilized, knowledge, and attitudes); and examining a predictor of sustainment guided by a conceptual framework (*i.e*., EPIS) [4].

The primary aims were to examine sustainment for clinicians two years following receiving training and consultation in cognitive-behavioral therapy (CBT) for youth anxiety [6], an evidence-based practice [7]. This time length was chosen given that it takes two to four years for programs to be institutionalized [8]. We selected penetration, the self-reported percentage of anxious youth treated with CBT [2], as our primary sustainment outcome given that it is an important implementation outcome [5] and to build upon the previous literature [2]. In an effort to broaden understanding of sustainment, we also examined sustainment of specific components of CBT and of two individual adopter characteristics from the EPIS framework [4]: knowledge of CBT and general attitudes regarding evidence-based practices (appeal, requirements, openness, and divergence). Although knowledge and attitudes are typically described as moderators of implementation, we were interested in examining knowledge and attitudes over time to see if initial changes in these constructs at the implementation phase maintained into the sustainment phase. Furthermore, consultation session attendance was examined as a moderator of the change in CBT knowledge and EBP attitudes across all time-points, given that consultation attendance was associated with an increase in treatment fidelity (*i.e*., skill; adherence) in the initial study [6].

## Method

### Participants

Of the 115 participants in the Beidas *et al*. (2012) study, 50 (43%) clinicians completed the sustainment study [6]. This response rate corresponds with similar studies [9]. Table 1 provides demographic information and background experience at baseline for the overall sample as well as for participants in the two-year follow-up study. Chi Square analyses comparing those who completed the follow-up versus those who did not indicated similar educational and licensure status. Completers were significantly less likely to be Hispanic/Latino compared to those who did not participate.

**Table 1:** Demographic and clinical background data in original sample and follow-up sample

**Variable**	**Overall sample**	**2-year follow-up**
	**(Beidas et al.,**[6]**;**	**Sample**
	**N = 115)**	**(N = 50)**
	n (%)	n (%)
Sex		
Male	11 (9.6%)	4 (8%)
Female	104 (90.4%)	46 (92%)
Race		
Caucasian	77 (67%)	37 (74%)
African American	15 (13%)	4 (8%)
Hispanic/Latino*	6 (5.2%)	0
Asian	5 (4.3%)	4 (8%)
Native American/Alaskan	1 (.9%)	0
Other	6 (5.2%)	2 (4%)
Missing	5 (4.3%)	3 (6%)
Educational Status		
Enrolled in graduate school	18 (15.7)	9 (18%)
Master’s degree	72 (62.6%)	32 (64%)
Doctor of philosophy	6 (5.2%)	3 (6%)
Doctor of psychology	5 (4.3%)	2 (4%)
Doctor of education	2 (1.7%)	2 (4%)
Medical doctor	6 (5.2%)	1 (2%)
Other degree	6 (5.2%)	1 (2%)
State Licensed	33 (28.7%)	15 (30%)
Previously treated anxious youth	58 (50.4%)	29 (58%)
	M (SD)	M (SD)
Age	35.93 (11.36)	35.09 (10.85)
Months of clinical experience	65.46 (82.38)	69.59 (86.85)
Identification with CBT	4.86 (1.68)	4.77 (2.02)
Caseload	19.48 (23.72)	18.65 (18.15)
Supervision per week^a^	1.57 (2.66)	1.29 (1.33)
Hour attendance at workshops	28.83 (76.18)	15.97 (19.54)
Previous supervision on CBT	0	0

*Note*. CBT = cognitive-behavioral therapy.

*Significant difference found between follow-up participants and non-participants.

^a^No additional details regarding type of supervision and/or topic of training workshops were gathered. However, it is worth noting that an exclusionary criterion for the Beidas *et al.* (2012) study was participating in more than 8 hours of previous training in CBT for child anxiety [6].

Table 2 provides information regarding study conditions, consultation participation, and training outcomes for the overall sample and two-year follow-up participants. No significant difference was found in training condition between follow-up participants and non-participants, χ^2^ (2, N *=* 115) = 3.70, p = 0.16. Follow-up participants completed significantly more consultation sessions than individuals who did not partake in the follow-up interview. With regard to training outcomes, no differences in levels of treatment fidelity (*i.e*., skill; adherence) were found between the follow-up participants and those who did not attend the follow-up at baseline, post-training, or post-consultation. No clinical outcomes directly pertaining to treated youth (*e.g*., diagnostic and/or symptom change) were gathered given our emphasis on implementation outcomes in the primary study (see Beidas *et al*., 2012; Proctor *et al*., 2010) [5],[6].

**Table 2:** Training condition, consultation participation, and training outcomes in original sample and follow-up sample

**Variable**	**Overall sample**	**2-year follow-up**
	**(Beidas et al.,**[6]**;**	**Sample**
	**N = 115)**	**(N = 50)**
	n (%)	n (%)
Training Condition		
Routine Training	41 (36%)	22 (44%)
Computer Training	34 (30%)	15 (30%)
Augmented Training	40 (35%)	13 (26%)
Trained to Skill Criterion		
Baseline	32 (28%)	19 (38%)
Post-training	73 (65%)	34 (68%)
Post-consultation	87 (85%)	41 (82%)
Trained to Adherence Criterion		
Baseline	7 (6%)	3 (6%)
Post-training	43 (38%)	23 (46%)
Post-consultation	62 (61%)	29 (58%)
	M (SD)	M (SD)
Consultation session attendance*	7.2 (3.2)	8.24 (2.26)

*Note*. Trained to criterion in skill equaled receiving a skill rating of 3.5 out 7 during a performance-based behavioral rehearsal. Trained to criterion in adherence equaled delivering 70% of cognitive-behavioral therapy components for youth anxiety during a performance-based behavioral rehearsal.

*Significant difference found between follow-up participants and non-participants.

At the time of follow-up, 84% of participants (N = 42) reported providing therapy to youth clients generally in the previous year. Participants reported a caseload of 0 to 75 child clients per week (M = 13.95, SD = 15.65). Of these clients, 0 to 100% involved anxious youth between the ages of 7 and 17 (M = 44.65, SD = 31.65). Of note, even though training specifically targeted youth between the ages of 7 and 17, follow-up participants reported implementing CBT with 52.68% of anxious youth younger than age 7 (SD = 44.66, range 0 to 100%) and with 84.08% of anxious adult clients (SD = 29.00, range 20% to 100%).

## Measures

### Clinician Demographics and Attitudes Questionnaire (CDAQ)

The CDAQ is a 15-item questionnaire that gathers background information (*e.g*., demographics, prior experience) [10].

### Evidence-Based Practice Attitude Sale (EBPAS)

The EBPAS is a 15-item self-report measure assessing participants’ attitudes toward the adoption and implementation of EBPs [11]. The EBPAS consists of four subscales: appeal, requirements, openness and divergence [11]. Appeal (Cronbach’s α = 0.80) refers to the extent to which a therapist would adopt a new practice if it was intuitively appealing. Requirements (Cronbach’s α = 0.90) refers to the extent to which a therapist would adopt a new practice if it was mandated. Openness (Cronbach’s α = 0.78) is the extent to which a therapist is generally open to trying new interventions. Divergence (Cronbach’s α = 0.59) is the extent to which a therapist perceives research-based treatments as lacking clinical utility [12]. In order to keep interpretation of the subscales consistent, Divergence scores were reverse-scored, such that higher scores indicated stronger beliefs in the usefulness of EBPs.

### Knowledge Test

This 20-item test measures knowledge of CBT for youth anxiety [10]. The test was developed and used in CBT training [13]. Three versions of the knowledge test were created to allow for repeated measures with minimal practice effects. Psychometric analyses were performed for the Beidas *et al*. (2012) and indicated a Cronbach’s α of 0.76 and Spearman-Brown split-half reliability of 0.69 [6]. Retest reliability was 0.86. Students trained in CBT for youth anxiety (M = 19.33, SD = 0.58) scored higher than untrained students (M = 13.71, SD = 2.75), (F (1, 9) = 11.51, p = 0.01), supporting the measure’s validity. For sample items, see Additional file 1.

### Adherence and Skill Checklist (ASCL)

This instrument measures treatment fidelity, including (a) adherence to the content of CBT for child anxiety and (b) skill in treatment delivery [10]. Adherence, which refers to the use of the procedures of a treatment protocol with a client [14], was assessed by coding the presence or absence of six core CBT competencies in treating child anxiety: (i) identification of somatic symptoms, (ii) identification of anxious cognition, (iii) relaxation, (iv) coping thoughts, (v) problem-solving, and (vi) positive reinforcement. Skill, which refers to the level of competence demonstrated by the clinician when delivering treatment [14], was assessed via a 7-point Likert scale ranging from 1 (not well) to 7 (very well). The ASCL measured adherence and skill demonstrated in 8-minute performance-based behavioral rehearsals [15], which involved clinicians preparing an anxious child (played by a trained undergraduate) for an exposure task. Coders (one doctoral level psychology graduate student, two post-undergraduate research assistants, and one honors undergraduate research assistant) were blind to condition and assessment time-point. Inter-rater reliability for the total adherence score was an ICC of 0.98. As a validity check, experienced CBT therapists reviewed the ASCL and rated it as accurately capturing the components of CBT for youth anxiety. See Additional file 2 for ASCL items.

### Identification and Treatment of Anxious Youth (ITAY)

Participants completed this 10-item self-report measure (Benjamin, Beidas, Edmunds, Cohen, & Kendall: Identification and Treatment of Anxious Youth, unpublished) at the completion of their consultation sessions during the Beidas *et al*. (2012) study to assess the application of the skills they had learned in the training [6]. Questions pertinent to the present study inquired about the number of anxious youth treated over the past three months and the number of anxious youth treated with CBT over the past three months. In this study, consistent with the definition provided by Proctor and colleagues (2011), we defined penetration as the percentage of anxious youth treated with CBT over the past three months (*i.e*., anxious youth treated with CBT divided by anxious youth treated overall) [5]. Specifically, we inquired about use of CBT generally, regardless of whether a particular CBT protocol was used (*e.g*., Coping Cat) [16]. A slightly modified version of the ITAY was administered during the two-year follow-up. Changes included administration in interview format, the addition of open-ended questions based on a similar interview guide [17], and inquiry about treatment use over the past year (see Additional file 3 for sample questions). For a qualitative analysis of therapists’ perspective on consultation gathered via the revised ITAY, please see Beidas *et al*. (2013) [18]. The investigator and one post-undergraduate research assistant served as interviewers. All interviews were audio-taped.

### Procedure

Before describing the procedures of the current study, a brief description of the original implementation trial is provided for context. For further details, please see Beidas *et al*. (2012) [6]. Participants were randomly assigned to one of the following three training modalities in CBT for child anxiety using equal allocation concealment at the level of training date: routine training (*i.e*., presentation detailing session by session treatment content following a specific treatment manual; Coping Cat [16]), computer training (*i.e*., self-paced training via computer program following a specific treatment manual; Coping Cat [16]), and augmented training (*i.e*., focused on core principles of CBT for child anxiety and incorporated experiential learning). All conditions taught CBT for child anxiety, an EBP for anxious youth [19]. Two of the conditions focused on a specific manualized CBT for child anxiety (Coping Cat) [16]. All participants were provided with Coping Cat materials following the training workshop [16],[20]. Importantly, no differences were found in outcomes following experimental manipulation of training strategies, suggesting that all were effective [6]. Following training, clinicians participated in weekly consultation sessions for three months, which included further didactics on CBT for youth anxiety and case discussion (see Edmunds *et al*., 2013, for description of consultation content) [21]. Table 3 details the assessment, intervention and consultation measurement schedule.

**Table 3:** Assessment, intervention, and consultation measurement schedule

**Measure**	**Pre-training**	**Post-training**	**Post-consultation**	**2-year follow-up**
Demographics	x			
Knowledge	x	x	x	x
Adherence	x	x	x	
Skill	x	x	x	
Attitudes	x	x	x	x
Org. Characteristics	x			
Penetration			x	x
CBT Components				x

*Note.* Pre-training assessment occurred at baseline, prior to training. Post-training assessment occurred immediately following training. Post-consultation assessment occurred immediately following 3 months of consultation. Two-year follow-up occurred 2 years following training. This table was adapted from Beidas *et al.* (2012) [6].

For the present study, all procedures were approved by Temple University's Institutional Review Board. We contacted all participants in the original study (N = 115) via an electronic newsletter to ascertain their interest in participating in a two-year-follow-up. A total of 50 consented to participate. Following consent, participants completed the EBPAS and the knowledge test via an online survey tool. The ITAY interviews were conducted individually via telephone with each of the participants at a time convenient for the participant with a study interviewer from October 2011 to April 2012. Interviews lasted approximately 45 to 60 minutes and were digitally recorded. Participants were compensated $10.00 for their participation.

### Data analysis

Descriptive analyses examined the average levels of penetration, CBT knowledge, attitudes toward EBPs, and components of CBT used at each time-point the measure was collected (see Table 3 for assessment schedule).

Penetration was measured across two time-points: immediately after receiving the training and consultation package and twoyears later. A paired samplet-test was conducted to compare the mean of penetration after participating in the implementation trial and two years following. Components of CBT used in treatment with anxious youth were measured at two-year follow-up only; descriptive analyses are presented.

Analyses pertaining to knowledge and attitudes were conducted using hierarchical linear modeling (HLM) given that these measures were administered across four time-points [22],[23]. The advantage of HLM is its ability to examine within-subject and between-subject change while accounting for the clustered nature of the data, particularly the change in outcomes across multiple time points within a single statistical model. In the present study, the Level 1 (within-subject) model analyzed the relationship between time and change in the CBT knowledge and attitudes toward EBPs of participants. The Level 2 (between-subject) model examined whether participation in consultation calls moderated the change in CBT knowledge and EBP attitudes across all time points. Participation in consultation sessions was examined as a potential moderator of change in knowledge and attitudes given that consultation attendance predicted higher levels of treatment fidelity at post-consultation [6]. Training condition was not examined as a potential moderator given that no significant changes in outcomes were found across conditions in the original study [6]. All predictor and moderating variables were grand-mean-centered.

## Results

Means and standard deviations for CBT knowledge, EBPAS scores, and penetration, as well as the number of participants at each assessment, are presented in Table 4. Means and standard deviations for CBT components are found in Table 5.

**Table 4:** Means and standard deviations of sustainment outcomes

**Measure**	**Pre-training**	**Post-training**	**Post-consultation**	**2-year follow-up**
Penetration				
*N*	-	-	54	41
M (SD)	-	-	79.48 (32.58)	84.39 (31.80)
CBT Knowledge				
*N*	115	115	92	50
M (SD)	15.14 (2.21)	17.22 (1.87)	17.63 (1.98)	16.20 (2.53)
EBPAS Requirements				
*N*	115	115	90	50
M (SD)	2.74 (1.03)	2.73 (1.15)	3.59 (.92)	2.85 (1.04)
EBPAS Appeal				
*N*	115	115	90	50
M (SD)	3.34 (.61)	3.45 (1.16)	4.26 (.54)	3.21 (.60)
EBPAS Openness				
*N*	115	115	90	50
M (SD)	3.17 (.58)	3.38 (.60)	4.09 (.61)	2.89 (.69)
EBPAS Divergence				
*N*	115	113	88	50
M (SD)	3.05 (.63)	3.11 (.57)	2.16 (.56)	3.22 (.59)

*Note.* Penetration rates were percentages. EBPAS = Evidence-Based Practice Attitude Scale.

**Table 5:** CBT components implemented at 2-year follow-up

**Item**	**M (SD)**	**Range**
Identification and management of somatic arousal	5.03 (1.09)	2-6
Identification and cognitive restructuring of self-talk	4.54 (1.25)	2-6
Problem-solving anxiety-provoking situations	4.56 (1.41)	0-6
Conducting imaginal exposures	2.49 (1.30)	0-4
Conducting behavioral/in vivo exposures	3.03 (1.75)	0-6
Utilizing positive reinforcement	5.03 (1.35)	2-6

*Note.* Rated on 7-point Likert scale from (*not at all*) to 6 (*extensively*) during Identification and Treatment of Anxious Youth (ITAY) interview at 2-year follow-up. CBT = cognitive behavioral therapy.

### Sustainment of penetration

A paired sample t-test showed no significant change in penetration (*i.e*., the proportion of anxious youth treated with CBT) from post-consultation (79.48%) to two-year follow-up (83.16%) (t (28) = −0.11, p = 0.91), suggesting that penetration of CBT for child anxiety was sustained.

### Sustainment of CBT components

Table 5 displays Likert ratings of specific CBT components sustained with anxious youth at the two-year follow-up on a scale from 0 (no use at all) to 6 (extensive use). The most extensively sustained components were (a) identification and management of somatic arousal, (b) identification and cognitive restructuring of anxious cognitions, (c) rewards, and (d) problem-solving. Exposure tasks in either imaginal or *in vivo* form were used least extensively.

### Sustainment of CBT knowledge

**Figure 1:**
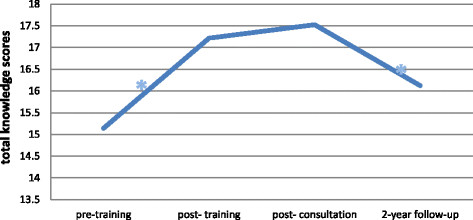
**Change in knowledge scores over time.** *indicates a significant change.

We examined the trajectories of knowledge of CBT principles across the four assessment points, with time measured categorically. Level of knowledge increased significantly from pre- to post- training, t (368) = 9.74, p < 0.001, from pre-training to post-consultation, t (368) = 9.86, p < 0.001, and from pre-training to two-year follow-up, t (368) = 3.08, p < 0.01. No increases in knowledge scores were found from post-training to post-consultation, t (368) = 1.36, p = 0.18. Knowledge significantly decreased from post-training to two-year follow-up, t (368) = −3.38, p = 0.001 and from post-consultation to two-year follow-up, t (368) = −4.58, p < 0.001 (see Figure 1).

### Sustainment of attitudes

#### EBPAS mean requirement scores

**Figure 2:**
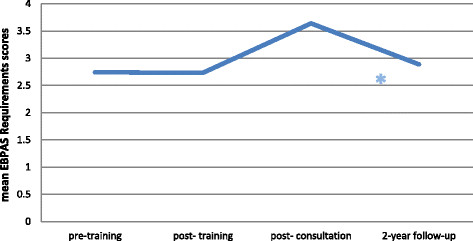
**Change in EBPAS mean requirement scores over time.** *indicates a significant change.

Mean EBPAS requirement scores did not significantly change from pre- to post-training, t (366) = −0.11, from pre-training to two-year follow-up, t (366) = 1.14, or from post-training to two-year follow-up, t (366) = 1.04 (allp’s > 0.05). Mean requirement scores increased from pre-training to post-consultation, t (366) =8.711, p < 0.001, from post-training to post-consultation, t (366) = 8.24, p < 0.001, and decreased from post-consultation to two-year follow-up, t (366) = −5.08, p < 0.001 (see Figure 2).

#### Sustainment of EBPAS mean appeal scores

**Figure 3:**
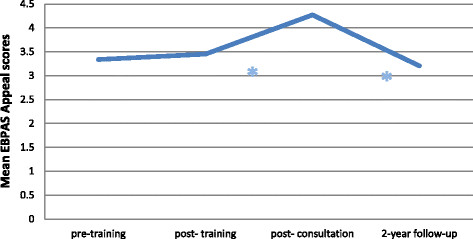
**Change in EBPAS mean appeal scores over time.** *indicates a significant change.

Mean EBPAS appeal scores did not significantly change from pre- to post-training, t (366) = 1.15, or from pre-training to two-year follow-up, t (366) = −1.54, (all p’s > 0.05). Mean appeal scores increased from pre-training to post-consultation, t (366) = 14.74, p < 0.001, from post-training to post-consultation, t (366) = 9.58, p < 0.001, decreased from post-training to two-year follow-up, t (366) = −2.33, p < 0.05, and from post-consultation to two-year follow-up, t (366) = −12.57, p < 0.001 (see Figure 3).

#### Sustainment of EBPAS mean openness scores

**Figure 4:**
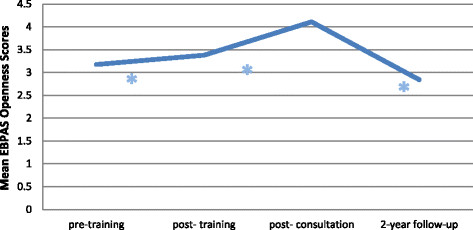
**Change in EBPAS mean openness scores over time.** *indicates a significant change.

Mean EBPAS openness scores significantly increased from pre- to post-training, t (366) = 4.77, p < 0.001, from pre-training to post-consultation, t (366) = 16.09, p < 0.001, and from post-training to post-consultation, t (366) = 13.19, p < 0.001, while openness scores decreased significantly from pre-training to two-year follow-up, t (366) = −3.65, p = 0.001, from post-training to two-year follow-up, t (366) = −5.90, p < 0.001, and from post-consultation to two-year follow-up, t (366) = −14.69, p < 0.001 (see Figure 4).

#### Sustainment of EBPAS mean divergence scores

**Figure 5:**
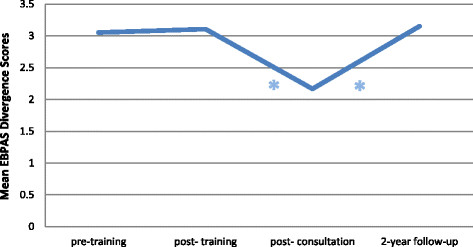
**Change in EBPAS mean divergence scores over time.** *indicates a significant change.

Mean EBPAS divergence scores did not significantly change from pre- to post-training, t (362) = 1.14, from pre-training to two-year follow-up, t (362) = 1.49, or from post-training to two-year follow-up, t (362) = 0.66 (allp’s > 0.05). Mean divergence scores significantly decreased from pre-training to post-consultation, t (362) = −15.44, p < 0.001, from post-training to post-consultation, t (362) = −18.39, p < 0.001, and significantly increased from post-consultation to two-year follow-up, t (362) = 15.58, p < 0 .001 (see Figure 5).

#### Moderation of outcomes by minutes spent in consultation sessions

Clinicians’ involvement in consultation sessions (minutes attended) was examined as a potential moderator of the change in CBT knowledge and EBP attitudes across all time-points. Minutes spent in consultation did not moderate the change in openness scores over time (all p’s > 0.05). However, this variable was identified as a significant moderator of all other examined outcomes across time. Significant findings are described below.

#### Knowledge

Mean minutes in consultation sessions moderated change in knowledge from pre-training to two-year follow-up, t (342) = 3.16, p < 0.01, and from post-training to two-year follow-up, t (342) = 3.50, p = 0.001, such that a positive deviation from the grand mean by one hour (60 minutes) was associated with increases in knowledge scores by 0.42 and 0.48 knowledge score points, respectively (see Table 6).

**Table 6:** Results of HLM models for change in CBT knowledge (total scores) from pre-training

	***Model***	
	**Empty model**	**Effect of minutes**
*Person-Level Variables*		
Initial Value (Intercept)	15.14 (.20)***	15.27 (0.21)***
Moderator	-	0.001 (0.001)
*Observation-Level Variables*		
Post	2.08 (0.21)***	1.98 (0.22)***
FU	2.39 (0.24)***	2.22 (0.27)***
2-year FU	0.98 (0.32)**	0.75 (0.30)*
*Cross-Level Interactions*		
Post X Moderator	-	-
FU X Moderator	-	0.0002 (0.003)
2-year FU X Moderator	-	0.007 (0.002)**

*Note.* Standard Error in parentheses. FU = follow-up. All level-2 variables were grand-mean centered. *p ≤ .05, **p ≤ .01, ***p ≤ .001.

#### Mean requirement scores

Mean minutes in consultation sessions moderated change in mean requirement scores from post-training to two-year follow-up, t (340) = 2.57, p < 0.05 and from post-consultation to two-year follow-up, t (340) = 2.51, p < 0.05, such that a positive deviation from the grand mean by one hour was associated with increases in mean requirements scores by 0.24 and 0.3 mean points, respectively (see Table 7).

**Table 7:** Results of HLM models for change in EBPAS attitude subscale mean scores

	***Model***	
	**Empty model**	**Effect of minutes**
***Requirements***		
*Person-Level Variables*		
Initial Value (Intercept)	2.74 (0.10)***	2.75 (0.10)***
Moderator	-	-.0006 (0.0007)
*Observation-Level Variables*		
Post	−0.009 (0.08)	−0.02 (0.08)
FU	0.90 (0.10)***	0.97 (0.10)***
2-year FU	0.14 (0.13)	−0.002 (0.16)
*Cross-Level Interactions*		
Post X Moderator		−0.001 (0.0007)*
FU X Moderator		−0.002 (0.001)
2-year FU X Moderator		0.003 (0.002)
***Appeal***		
*Person-Level Variables*		
Initial Value (Intercept)	3.34 (0.06)***	3.37 (0.05)***
Moderator	-	0.0001 (0.0004)
*Observation-Level Variables*		
Post	0.11 (0.10)	0.12 (0.11)
FU	0.93 (0.06)***	0.93 (0.07)
2-year FU	−0.13 (0.09)	−0.25 (0.10)**
*Cross-Level Interactions*		
Post X Moderator	-	−0.0001 (0.0009)
FU X Moderator	-	−0.0005 (0.0007)
2-year FU X Moderator	-	0.003 (0.001)*
***Openness***		
*Person-Level Variables*		
Initial Value (Intercept)	3.17 (0.05)***	
Moderator	-	
*Observation-Level Variables*		
Post	0.20 (0.44)***	
FU	0.94 (0.06)***	
2-year FU	−0.30 (0.09)**	
*Cross-Level Interactions*		
Post X Moderator	-	
FU X Moderator	-	
2-year FU X Moderator	-	
***Divergence***		
*Person-Level Variables*		
Initial Value (Intercept)	3.05 (0.06)***	3.06 (0.06)***
Moderator	-	−0.001 (0.0005)
*Observation-Level Variables*		
Post	0.05 (0.04)	0.07 (0.05)
FU	−0.89 (0.06)***	−0.89 (0.06)***
2-year FU	0.10 (0.07)	−0.01 (0.06)
*Cross-Level Interactions*		
Post X Moderator	-	0.001 (0.001)
FU X Moderator	-	0.0001 (0.001)
2-year FU X Moderator	-	0.002 (0.001)***

*Note.* Models with nonsignificant effects were not included in the table. Standard Error in parentheses. FU = follow-up; All level-2 variables were grand-mean centered. *p ≤ .05, **p ≤ .01, ***p ≤ .001.

#### Mean appeal scores

Mean minutes in consultation sessions moderated change in mean appeal scores from pre-training to two-year follow-up, t (340) = 2.13, p < 0.05, from post-training to two-year follow-up, t (340) = 3.24, p < 0.01, and from post-consultation to two-year follow-up, t (340) = 2.59, p = 0.01, such that a positive deviation from the grand mean by one hour was associated with increases in mean appeal scores by 0.18, 0.24, and 0.18 mean points, respectively (see Table 7).

#### Mean divergence scores

Mean minutes in consultation sessions moderated change in mean divergence scores from pre-training to two-year follow-up, t (336) = 3.96, p < 0.001, from post-training to two-year follow-up, t (336) = 5.73, p < 0.001, and from post-consultation to two-year follow-up, t (336) = 3.24, p < 0.01, such that a positive deviation from the grand mean by one hour was associated with increased mean divergence scores 0.12, 0.18, and 0.12, mean divergence points, respectively (see Table 7).

## Discussion

The present study examined sustainment of CBT for anxious youth two years following participation in an implementation trial using educational implementation strategies (*i.e*., training and consultation). Guided by the EPIS framework, the sustainment of two individual adopter characteristics (*i.e*., knowledge and attitudes) was also examined [4]. Time spent in consultation sessions was examined as a potential moderator of change in knowledge and attitudes over time based on previous literature. Given that participation was largely driven by individual choice, consultation participation can also be considered an individual adopter characteristic [4]. Findings indicate sustainment of penetration rates from post-consultation to follow-up although not all intervention elements were equally delivered. Therapists reported low usage rates of specific components of the treatment, specifically exposure tasks, the active ingredient of CBT for child anxiety [24]. Although knowledge scores at follow-up were significantly higher than at baseline, maximal gains in knowledge achieved by post-consultation were not sustained. Attitudinal improvements toward EBPs following training and consultation were also generally not sustained. Time spent in consultation significantly moderated changes in knowledge and attitudes toward EBPs, suggesting an important mutable target for implementation and sustainment strategies moving forward. Taken together, these results suggest important implications for future implementation endeavors.

Self-reported penetration of CBT with anxious youth was sustained from post-consultation (79%) to follow-up (83%). Fidelity was not measured at the two-year follow-up, but therapists were asked to report on components of CBT for child anxiety that they had used with anxious youth. These self-report ratings suggest that CBT may have not been implemented with high fidelity. Clinicians reported extensively implementing identification and management of somatic arousal and anxious cognitions, rewards, and problem-solving. Exposure tasks, a key component of CBT for anxiety, were implemented much less extensively. This is concerning given that the exposure tasks are critical and are viewed as core components of the intervention [24]. When considering the treatment components needed to attain fidelity, even with ‘flexibility’, [25], exposure tasks are required [26]. In light of these findings, implementation efforts using training and consultation as implementation strategies may benefit from spending additional time discussing the importance and application of exposure tasks, including how clinicians might tailor exposures within their organization. Of note, approximately 30% of the consultation sessions in the present study focused on exposure tasks, suggesting that spending more time in consultation on exposure is necessary [21], although the specific amount of time needed in training and consultation to dedicate to exposure remains unknown.

Knowledge increases throughout the training and consultation phase were not sustained at two-year follow-up, although knowledge remained higher than at baseline. In order to identify contextual factors that contribute to sustainment, and in accord with the EPIS framework, an individual adopter characteristic (*i.e*., time spent in consultation) was examined as a potential moderator given previous findings (see Beidas *et al*., 2012) [4],[6]. Greater participation in consultation resulted in greater sustainment in knowledge at the follow-up period. This finding is consistent with previously reported findings and provides further evidence of the benefits of consultation following workshop training in order to improve and sustain knowledge [6]. It is worth noting that follow-up participants spent more time in consultation than non-participants. Thus, higher than average participation in consultation within a group already evidencing greater consultation than the general group appeared to contribute to positive results, further highlighting consultation as an important educational implementation strategy.

Disappointingly, improvements in attitudes gained through the training and consultation did not sustain over time. The pattern observed across two of the four attitudinal subscales measured by the EBPAS (appeal, requirements) suggested that attitudinal gains made by participating in training and consultation were not maintained. A similar pattern emerged with regard to openness to new practices, but surprisingly, not only were gains not maintained, but clinicians exhibited less openness to new practices at follow-up when compared to baseline. Belief in the utility of EBPs exhibited a different pattern. This attitude did not change from baseline to post-training, decreased from post-training to post-consultation, and then increased to baseline levels from post-consultation to follow-up. There is little literature to ground these findings within because few studies have investigated changes in attitudes over this long of a period (*i.e*., sustainment). Many studies measure attitudes pre- and post-training [27], and, typically, attitudes do improve following training as observed from baseline to post-consultation in this study (see Beidas & Kendall, 2010) [28]. However, this study demonstrates that new information is gleaned through measurement of attitudes at the sustainment time-point. With regard to the curious finding that openness to new practices dropped at follow-up when compared to baseline, one explanation may be that therapists who were initially very open to new practices became aware that a significant time investment (*i.e*., training and ongoing consultation) is necessary to gain mastery in a new practice, making them less open to new practices in the future. It is also possible that clinicians encountered difficulty implementing exposures on their own during the follow-up period, which may have resulted in less openness to CBT given its emphasis on exposures. With regard to the finding that belief in the utility of EBPs increased from post-consultation to follow-up, it is possible that after having two years to apply CBT for youth anxiety, therapists had more time to see the potential benefits of the treatment and generalized this to other EBPs.

Consultation emerged as an important moderator with regard to both knowledge and attitudes toward EBPs. As noted above, attitudinal increases from training and consultation were not maintained at follow-up. However, number of minutes spent in consultation impacted sustainment of attitudes. In other words, spending more time in consultation resulted in higher attitudes with regard to requirements, appeal, and divergence as measured by the EBPAS. It may be that greater exposure to CBT for child anxiety through consultation highly impacted therapists’ attitudes toward whether or not they would be likely to adopt EBPs if they were required to, found them appealing, or believed in their utility. Importantly, the results from this study suggest that consultation is an implementation strategy that can be leveraged to change both knowledge and attitudes towards EBPs over time*.* In other words, the impact of three months of consultation provided in relatively low dosage (maximum of 1 hour a week for 12 weeks) can be continued to provide a return on investment up to twoyears later. It is important to also observe if consultation has a similar impact on fidelity and/or client outcomes in future studies. Also, given that consultation participation relies on individual choice to an extent, and thus can be considered an individual adopter characteristic, it is important to understand what factors predict greater participation in consultation.

This study adds to the limited literature regarding the sustainment of penetration, knowledge and attitudes over time. Strengths of the study include the two-year span of time examined as well as the inclusion of an individual adopter characteristic (*i.e*., consultation participation) as a potential moderator of sustainment. Additionally, this study looked at numerous sustainment outcomes, including outcomes not commonly examined (*e.g*., knowledge and attitudes). However, limitations should be noted. Despite best efforts, only 43% of the original sample participated. A lower proportion of Hispanics/Latinos participated in the follow-up study as compared to the original study. Also, a difference in consultation session attendance was found across samples. Thus, it is possible that findings from the current study do not generalize to the full sample. Given the specific focus on CBT for youth anxiety, it is possible that findings do not generalize to other interventions [29]. Other limitations pertain to measures used, including investigator-created measures and the use of self-report.

Additionally, the current study only examined one individual adopter characteristic as a potential moderator of sustainment, whereas the EPIS framework encourages examination of numerous additional inner context factors, intervention characteristics, and outer context factors [4]. Inner context factors include intra-organizational and individual adopter characteristics, whereas outer context factors include characteristics of the service environment, inter-organizational environment, and consumer support/advocacy [4]. Also, given that participants were not randomized to amounts of consultation attendance, it is possible that attendance was confounded by motivation; participants who spent more time in consultation may have had higher motivation as well as greater knowledge and more positive attitudes. Furthermore, due to time and resource constraints, the follow-up study did not include an assessment of treatment fidelity, preventing conclusions regarding the sustainment of this important implementation outcome. Future work on sustainment is needed to address these limitations. To gain a better understanding of change over time, future investigations should study sustainment over even longer periods of time. With regard to measurement, future work should incorporate multiple outcome measures, including observational fidelity (*e.g*., coded therapy sessions) and client outcomes. Examination of potential moderators of sustainment, informed by an ecological framework, such as EPIS, will allow for identification of mutable targets to improve implementation and sustainment efforts [4].

## Conclusion

The field has made gains regarding the development and implementation of EBPs [19],[30]. Despite advancements, much work is left to be done, particularly around sustainment [2],[3]. The present study adds to this limited literature on sustainment by demonstrating that although clinicians sustained self-reported penetration, they did not deliver the treatment with intended components, specifically exposure tasks. Further, knowledge and improved attitudes toward EBPs failed to fully sustain over the two-year follow-up period. However, the silver lining to these discouraging results further corroborates the importance of ongoing consultation following initial training as an important implementation strategy in order to promote and sustain knowledge and attitudes over time.

## Competing interests

The authors declare that they have no competing interests.

## Authors’ contributions

JME conceptualized the follow-up study, contributed to data collection and coding, and was the predominant contributor to this article. RSB conceptualized the training study and follow-up study, contributed to the data collection, and was a key contributor to this article. KLR contributed to coding, completed data analyses, and assisted with interpretation of results. VAR and DMB contributed to coding and assisted with interpretation of results. PCK assisted in conceptualization of both the original training study and the follow-up study and assisted in interpretation of results. All authors read and modified drafts and approved the final manuscript.

## Additional files

## Supplementary Material

Additional file 1: **Sample questions from Knowledge Test.** Includes a few sample items from the measure assessing knowledge of cognitive-behavioral therapy for youth anxiety. Click here for file

Additional file 2: **Adherence and Skill Checklist.** Includes the checklist used to code treatment fidelity during behavioral rehearsals. Click here for file

Additional file 3: **Sample questions from Identification and Treatment of Anxious Youth - Revised.** Includes a few sample items from the revised version of the Identification and Assessment of Anxious Youth (ITAY) measure that was delivered in interview format at the two-year follow-up. Click here for file
