# Supplementary material for: Sustaining clinician penetration, attitudes and knowledge in cognitive-behavioral therapy for youth anxiety
Source: Implement Sci. 2014 Jul 17;9:89. doi: 10.1186/s13012-014-0089-9 (PMC4223397; doi:10.1186/s13012-014-0089-9)
Supplement: Additional file 1: — Sample questions from Knowledge Test. Includes a few sample items from the measure assessing knowledge of cognitive-behavioral therapy for youth anxiety. [file s13012-014-0089-9-S1.pdf]

Sample Questions from Knowledge Test

1. Identify which one is not a thinking trap.
    a. The avoider: Staying away from situations you think are scary without trying first
    b. The mind-reader: Jumping to conclusions about a person/thing/situation without the facts
    c. The shoulds: I should always be perfect, I shouldn't make mistakes
    d. The gooder: Only seeing the positive and not looking at the bad things in the situation

2. Progressive muscle relaxation is composed of:
    a. Tensing and relaxing parts of your body
    b. Taking a nap
    c. Deep breathing
    d. A and C

3. Which of the following is most true with regard to coping modeling?
    a. Mastery modeling is preferred over coping modeling
    b. Coping modeling increases the similarity between the observer and the model
    c. Coping modeling includes initial difficulties (like those of the client), a strategy to overcome the difficulty, and then success
    d. Both b and c are correct

4. Which of the following is not an action a youth can take to help themselves when they are feeling anxious?
    a. Problem solving
    b. Coping thoughts
    c. Deep breathing
    d. Progressive muscle relaxation
    e. Getting mom to help them get out of the situation

5. When the exposure portion of the treatment begins, the therapist should
    a. Display anxiety about exposure tasks
    b. Show confidence in the child
    c. Introduce the most frightening stimulus to the child
    d. Ask the parents for guidance
