# Supplementary material for: Sustaining clinician penetration, attitudes and knowledge in cognitive-behavioral therapy for youth anxiety
Source: Implement Sci. 2014 Jul 17;9:89. doi: 10.1186/s13012-014-0089-9 (PMC4223397; doi:10.1186/s13012-014-0089-9)
Supplement: Additional file 2: — Adherence and Skill Checklist. Includes the checklist used to code treatment fidelity during behavioral rehearsals. [file s13012-014-0089-9-S2.pdf]

Adherence and Skill Checklist

<u>Adherence Checklist</u>

1. Preparing the child for the exposure

   a. Feeling Frightened/Somatic Symptoms of Anxiety

      i. Did the therapist help the child identify what physical feelings they might experience during the exposure (e.g. stomach ache)?

      Yes No

   b. Expecting Bad Things to Happen/Anxious Cognitions

      i. Did the therapist help the child identify what anxious thoughts they might have during the exposure (e.g., I might make a mistake)?

      Yes No

   c. Attitudes and Actions that can Help

      i. Did the therapist mention or have the child practice deep breathing and/or progressive muscle relaxation to cope with anxious bodily feelings (e.g., belly breathing, squeezing lemons)?

      Yes No

      ii. Did the therapist help the child generate a coping thought as a way to cope with anxious thoughts (e.g., I can do it, Even if I make a mistake, I can get through it)?

      Yes No

      iii. Did the therapist walk the child through problem-solving possible problems that may come up during the exposure (e.g., what to do if you make a mistake – walk away, ask the question again)?

      Yes No

   d. Rewards

      i. Did the therapist plan a reward with the child for facing their fear (e.g., special time with therapist, ice cream)?

      Yes No

<u>Core Adherence: /6</u>

2. Other general considerations

a. Did the therapist ask the child's anxiety rating (e.g., SUDS rating) at any point (e.g., What is your number, fear rating, etc.)?

Yes No

b. Did the therapist use cognitive restructuring techniques to help the child come up with a coping thought (e.g., How likely is it that you will mess up? Has that ever happened to anyone you know before? What is the worst that can happen)?

Yes No

c. Did the therapist have the child imagine engaging in the exposure (e.g., practicing calling the grandmother?)

Yes No

d. Was the therapist a coping model (i.e., gave an example of when they felt anxious and how they coped?)

Yes No

Additional Adherence factors: ____/4

TOTAL ADHERENCE SCORE (CORE+ADDITIONAL): _______/10

Skill Ratings

1. How skillful was the clinician's performance in preparing the child for the exposure using the cognitive-behavioral framework? (i.e. how well did the individual show the skills they learned?)

1	2	3	4	5	6	7
Not well						Very Well

2. How coherent was the presentation of the exposure task? (i.e. how all over the place was the therapist, how organized were they, did they follow the organization outlined in the adherence section)

1	2	3	4	5	6	7
Not coherent						Very coherent
