# Supplementary material for: Sustaining clinician penetration, attitudes and knowledge in cognitive-behavioral therapy for youth anxiety
Source: Implement Sci. 2014 Jul 17;9:89. doi: 10.1186/s13012-014-0089-9 (PMC4223397; doi:10.1186/s13012-014-0089-9)
Supplement: Additional file 3: — Sample questions from Identification and Treatment of Anxious Youth - Revised. Includes a few sample items from the revised version of the Identification and Assessment of Anxious Youth (ITAY) measure that was delivered in interview format at the two-year follow-up. [file s13012-014-0089-9-S3.pdf]

Sample Questions from Identification and Treatment of Anxious Youth - Revised

Treatment of Anxious Youth

1. During this last year, what has your average child/adolescent caseload been per week?

2. What percentage of your average weekly child/adolescent caseload involves anxious youth between the ages of 7 and 17?

3. Of the anxious youth you treated, what percentage did you treat with CBT?

4. Rate how extensively, on average, the following components of CBT for youth anxiety were used with all anxious youth you treated:

0	1	2	3	4	5	6
No use		Some use		Considerable use		Extensive use

   a. Identification and management of somatic arousal

   b. Identification of anxious thoughts/self-talk and cognitive restructuring/changing self-talk

   c. Problem-solving

   d. Imaginal exposures

   e. Behavioral exposures

   f. Positive reinforcement
